# Census of 1850

**Published:** 1855-10

**Authors:** 


### CENSUS OF 1850.

| | | | | | |
|---|---|---|---|---|---|
| Males | 6,546,753 | Females | 6,558,136 | Excess of Fem. | 11,000 |
| Free col'd | 190,000 | " | 210,000 | " col. " | 20,000 |
| Slaves | 1,602,525 | " | 1,601,778 | " " " | 700 |

The above includes only persons, the nativities of whose states were known. New York state contains one-eighth of the whole population of the Union, Pennsylvania one-tenth.

There are nearly four million dwelling houses, making about five persons to each house.

The smallest town in the Union is Liberty, in Keokuk county, Illinois, having five inhabitants. Averill in Vermont has seven.

Clergymen, 27,000. Lawyers, 24,000. Editors, 13,000.
Artists 2,000. Butchers, 18,000, not including doctors!
Blacksmiths, 100,000. Churches, 35,000.

There are five million milch cows, not including town pumps, nor the Hudson River.

Persons going to school, 4,127,000. Teachers, 104,260.

There are in the United States over a million of white persons who cannot read or write, one-fifth of these are foreigners.

It is an inquiry of much interest why, according to the above, every twenty free colored persons give one extra female, while it requires 1191 whites to give one extra female, and 4578 slaves to give one extra female? Does it mean that there is a natural connection between monogamy and a life of steady labor with plain substantial food? Does it mean that Providence designed that a man should work, live plainly, with one wife, and have health, competence, and a *rational old age*, there being a thousand per cent more crazy white people than colored slaves?

